# A Pain in the Neck: Lessons Learnt from Genetic Testing in Fetuses Detected with Nuchal Fluid Collections, Increased Nuchal Translucency versus Cystic Hygroma—Systematic Review of the Literature, Meta-Analysis and Case Series

**DOI:** 10.3390/diagnostics13010048

**Published:** 2022-12-23

**Authors:** Gioia Mastromoro, Daniele Guadagnolo, Nader Khaleghi Hashemian, Laura Bernardini, Antonella Giancotti, Gerardo Piacentini, Alessandro De Luca, Antonio Pizzuti

**Affiliations:** 1 Department of Experimental Medicine, Sapienza University of Rome, 00185 Rome, Italy; 2 Department of Laboratory Medicine, Fatebenefratelli Isola Tiberina–Gemelli Isola, 00186 Rome, Italy; 3 Cytogenetics Unit, Casa Sollievo della Sofferenza Foundation, San Giovanni Rotondo, 71013 Foggia, Italy; 4 Department of Maternal and Child Health and Urological Sciences, Sapienza University of Rome, 00161 Rome, Italy; 5 Department of Neonatology and Fetal Cardiology, Fatebenefratelli Isola Tiberina–Gemelli Isola, 00186 Rome, Italy; 6 Medical Genetics Division, Fondazione IRCCS Casa Sollievo della Sofferenza, 71013 San Giovanni Rotondo, Italy

**Keywords:** increased nuchal translucency, cystic hygroma, molecular testing, prenatal diagnosis, array, RASopathies

## Abstract

Fetal Nuchal fluid collections can manifest with two distinct presentations attributable to the same phenotypic spectrum: increased nuchal translucency (iNT) and cystic hygroma. The prenatal detection of these findings should prompt an accurate assessment through genetic counseling and testing, including karyotype, chromosomal microarray analysis (CMA) and multigene RASopathy panel. We performed a systematic review of the literature and meta-analysis, to calculate diagnostic yields of genetic testing in fetuses with iNT and cystic hygroma. We compared the results with a cohort of 96 fetuses with these isolated findings. Fetuses with isolated NT ≥ 2.5 mm showed karyotype anomalies in 22.76% of cases and CMA presented an incremental detection rate of 2.35%. Fetuses with isolated NT ≥ 3 mm presented aneuploidies in 14.36% of cases and CMA had an incremental detection rate of 3.89%. When the isolated NT measured at least 3.5 mm the diagnostic yield of karyotyping was 34.35%, the incremental CMA detection rate was 4.1%, the incremental diagnostic rate of the RASopathy panel was 1.44% and it was 2.44% for exome sequencing. Interestingly, CMA presents a considerable diagnostic yield in the group of fetuses with NT ≥ 3.5 mm. Similarly, exome sequencing appears to show promising results and could be considered after a negative CMA result.

## 1. Introduction

Fetal body fluid collections occur when the rate of interstitial fluid production by capillary ultrafiltration exceeds the rate of interstitial fluid return to the circulation via lymphatic vessels [1]. The characteristics of prenatal microcirculation and lymphatic system make fetuses more prone to develop interstitial fluid accumulation, due to the higher capillary permeability, the higher interstitial compartment compliance, and the greater influence of venous pressure on lymphatic return. Conditions that alter homeostatic mechanisms can perturb the balance of interstitial fluid movement.

Currently, there is still confusion regarding the etiopathogenesis and the possible associations with chromosomal, genomic, and monogenic conditions following the detection of nuchal fluid collections. These can present as two distinct nosological entities, increased nuchal translucency (iNT) and cystic hygroma. Some authors do not consider the need to distinguish the two ultrasound pictures relevant, as often in pregnancies with these findings the same management is indicated [2]. However, iNT and cystic hygroma appear to present different risks, which must be provided in the genetic counseling, therefore the distinction between the two entities can even play a role in the couple’s decisions regarding the current pregnancy.

The term “nuchal translucency” represents the sonographic measurement of the widest thickness of the physiological collection of fluid under the skin behind the fetal neck that is measured when the crown-rump length ranges between 45 and 84 mm, between 11 and 13 weeks of gestational age (wga) [3,4,5]. This collection has a transient nature and is not considered a structural anomaly. NT is measured in a midsagittal view of the fetal face, defined by the presence of the echogenic tip of the nose and rectangular shape of the palate anteriorly, the translucent diencephalon in the center and the nuchal membrane posteriorly [3,4,5]. Physiological NT and iNT should not present septations in this view [6].

NT ≥ 99th centile (≥3.5 mm), found in around 1% of pregnancies, is associated with chromosomal anomalies, monogenic conditions [7], heart malformations and other malformations in euploid fetuses [8]. NT  ≥  3 mm [9], found in around 5% of screened fetuses [10], is considered a high risk factor for aneuploidies, while the interpretation of the 2.5–2.9 mm NT is controversial.

Cystic hygroma represents a developmental anomaly of the lymphatic system, characterized by a protein-rich fluid [11] located in the nuchal region, behind and around the fetal neck, which can extend the length of the fetus and show septations. It is found in 1:285 fetuses [12] and it arises from the jugular-lymphatic obstruction and can progress determining hydrops. Cystic hygroma is associated with poor prognosis. However, since it is not possible to use the septation to distinguish cystic hygroma and increased nuchal translucency [2], in the literature the criterion used to attribute the ultrasound finding to one or the other entity is not always clear.

These conditions can occur as isolated, or in association with fetal structural anomalies [2,12]. When further anomalies are present, in some cases the association can be ascribed to the co-occurrence of two or more anomalies due to the same underlying cause (e.g., specific genetic disorders) [2,12]. In other cases, the primary anomaly, such as a cardiac malformation or a skeletal dysplasia, might hinder lymphatic development and function, secondarily resulting in a fetal nuchal fluid collection. In these circumstances, the lymphatic anomaly might be regarded as a secondary deformation rather than a primary developmental disorder.

The detection of iNT or cystic hygroma should prompt genetic counseling. Anamnestic reassessment, further sonographic evaluations and cytogenetic and molecular testing play a decisive role in management of fetuses with these findings. It should be noted that cases that are apparently isolated at the time of counseling might be later detected with additional anomalies which were not evident at primary examinations. Standard karyotype and chromosomal microarray analysis (CMA) are usually requested, even if when the NT is in the normal range but above the 95th centile some authors propose non-invasive prenatal screening [13]. In other cases, when cytogenetic testing yields negative results, Next Generation Sequencing (NGS) RASopathy panel or clinical exome sequencing can be proposed to the couple [14].

RASopathies are a group of conditions determined by pathogenic variants in genes involved in the Ras/mitogen-activated protein kinase (RAS-MAPK) signaling pathway, that can be prenatally suspected when sonography identifies iNT, cystic hygroma, pleural/pericardial effusions, polyhydramnios or some subtypes of cardiac malformations, even if these are nonspecific findings [15]. According to the literature, an extensive multigene panel should be offered when an RASopathy is suspected in the prenatal setting [15].

Conflicting results in the literature may be explained by both different NT distributions and parameters used to select the cohort.

We performed a systematic review of the literature and meta-analysis, in order to calculate diagnostic yields of genetic testing in fetuses with iNT, and compared the results with fetuses detected with cystic hygroma. We also collected a dicentric case series of fetuses with these isolated findings that underwent karyotype, CMA and RASopathy panel sequentially.

## 2. Materials and Methods

### 2.1. Systematic Review of the Literature and Meta-Analysis

The research was conducted following PRISMA guidelines [16]. We searched the Pubmed database (https://pubmed.ncbi.nlm.nih.gov/), accessed on 21 October 2022, from 2002, for (“fetus” or “fetuses” or “foetus” or “foetuses” or “fetal” or “foetal” or “fetalis” or “foetalis” or “prenatal” or “pre-natal” or “antenatal” or “ante-natal”) and (“hygroma” or “cystic hygroma” or “septated cystic hygroma” or “NT” or “nuchal translucency” or “nuchal thickness”) and (“karyotype” or “karyotyping” or “cytogenetic analysis” or “molecular cytogenetic” or “molecular cytogenetics” or “CMA” or “chromosomal microarray” OR “chromosomal microarrays” or “chromosomal array” or “chromosomal arrays” or “CGH array” or “comparative genomic hybridization” or “SNP array” or “microdeletion” or “microduplication” or “CNV” or “CNVs” or “copy number variant” or “copy number variants” or “copy number variation” or “copy number variations” or “WES” or “CES” or “Exome Sequencing” or “RASopathy” or “RASopathies”). All titles and abstracts were examined. Only papers with full text in English were included. Papers discussing or describing the prenatal diagnostic application of karyotyping, chromosomal microarray, RASopathies molecular testing and/or exome sequencing in iNT and/or cystic hygroma were retained, and full texts were examined. Papers reporting the diagnostic yields of at least one of such techniques in cases of iNT and/or cystic hygroma were deemed eligible for quantitative analysis. A further quantitative analysis was performed for papers reporting the postnatal outcomes of euploid fetuses from these cohorts. iNT cohorts and cystic hygroma cohorts were analyzed separately. Only singleton pregnancies were retained. Cases with post-mortem diagnosis were excluded. Papers in which cystic hygroma cases were included in NT cohorts were excluded if such cases could not be separated from the rest of the series. Papers in which post-mortem cases and twin pregnancies could not be removed from the count were secondarily excluded. The cases were classified as “apparently isolated”, “associated”, or “unspecified”. The classification was retrieved from the reported association status of the primary anomaly at the time of referral. We classified as “apparently isolated” the cases that did not present additional structural or functional anomalies during the first and second trimester and before genetic assessment, regardless of whether other anomalies were later identified or not. Cases in which additional anomalies were identified during the first or second trimester ultrasound scan and before genetic testing were classified as “associated”. These categories were adopted as they represent the two possible initial presentations for cases undergoing genetic counseling and invasive diagnostic procedures in clinical practice. If information on associated anomalies at the time of initial assessment was not provided or could not be retrieved from the cohorts, the cases were classified as “unspecified”. If specified, the values of NT (in centiles or millimeters) of each cohort were reported. For each category, we annotated the diagnostic yield of standard karyotyping, the incremental diagnostic yield of CMA after negative karyotyping, and the subsequent incremental diagnostic yield of RASopathy testing and exome sequencing. For the purpose of these scores, aneuploidies and Copy Number Variations (CNVs) > 10 Mb in extension identified with molecular techniques performed as first-tier testing were considered karyotype-detectable. Only variants classified as Pathogenic or Likely Pathogenic (represented together by the acronym P/LP) according to the American College of Medical Genetics (ACMG) guidelines [17,18] and relevant to the phenotype were considered as diagnostic. When available, we also gathered Variants of Uncertain Significance (VUS) and incidental findings. In addition, pregnancy and postnatal outcomes were recorded for euploid fetuses, specifying whether CNVs or monogenic syndromes were excluded or not. We annotated the rates of miscarriages, intrauterine deaths, live births, perinatal death, major childhood morbidity and apparently isolated intellectual disability. 

We then performed a meta-analysis of the diagnostic rates of cytogenetics and molecular prenatal diagnostics in iNT and cystic hygroma. Cases from eligible papers were divided by initial finding (iNT or Cystic Hygroma), association with other anomalies excluding soft markers (Unspecified, Apparently Isolated, Associated), and by the undertaken tests (Standard Karyotyping, CMA, RASopathies testing, Exome Sequencing). Cases with iNT were divided in ranges as 2.5–2.9 mm, 2.5–3.5 mm, 3.0–3.5 mm, ≥2.5 mm, ≥3.0 mm, ≥3.5 mm. The 2.5 mm, 3.0 mm and 3.5 mm cut-offs were chosen as they represent different accession criteria for invasive testing in different countries either in clinical or research settings. Cases belonging to the same category were pooled from reference papers. We then scored the diagnostic yield of standard karyotyping in each class, then incremental diagnostic yield of CMA in karyotype-negative cases, subsequently the further incremental yield of RASopathy testing in karyotype- and CMA-negative cases, and as a final tier the yield of ES. For the purpose of this review, all chromosomal number anomalies and chromosomal imbalances ≥ 10 Mb were considered karyotype-detectable even if originally identified with molecular techniques. Following the same principle, variants in RASopathy genes identified by exome sequencing were classified as RASopathy-panel-detectable. The yield was calculated for each category and tested as “pooled number of cases with a Pathogenic or Likely Pathogenic (P/LP) variant”/“pooled number of recruited cases”. Standard deviations and 95% confidence intervals were scored with the = STDEV.S and = CONFIDENCE functions on Microsoft Excel (Office 365).

### 2.2. Fetal Cohort

We collected a cohort of 96 fetuses detected with iNT (≥2.5 mm) or cystic hygroma from Policlinico Umberto I and Fatebenefratelli Isola Tiberina-Gemelli Isola Hospitals. After genetic counseling, each couple was offered genetic testing after invasive procedures (chorionic villus sampling or amniocentesis). After informed consent was collected, each sample underwent karyotyping. In cases with normal fetal karyotype, CMA was offered to the couple. In the same way, cases with negative CMA results were offered the NGS RASopathy panel. The fetuses did not show structural anomalies when they underwent invasive procedure and genetic testing.

We excluded from our cohort fetuses diagnosed with malformations at the time the genetic tests were performed (in order to make the data comparable to each other) and families with recurrent iNT or cystic hygroma (with higher suspicion of a monogenic condition). We included fetuses detected with soft markers, noting them.

For karyotyping, the sample was seeded on culture medium with CHANG for 10–15 days and metaphases obtained after treatment with colchicine were G-banded following standard procedures. At least 16 colonies were analyzed.

Genomic screening for CNVs was performed using the Cytoscan HD (Thermo Fisher Scientific, Waltham, MA, USA) or the 180 K oligonucleotide array (Agilent Technologies, Waldbronn, Germany) microarray platform, following the manufacturer’s instructions and using the ChAS (Thermo Fisher Scientific) or Cytogenomics (Agilent Technologies) analysis software, respectively. Both microarray platforms had 75 Kb effective resolution. Rearrangements were confirmed by real-time quantitative PCR on fetal and parental DNA. In accordance with the guidelines of the American College of Medical Genetics (ACMG), the detected CNVs were classified as pathogenic (P), probably pathogenic (LP), variants of uncertain significance (VUS), probably benign (LB) or benign (B) [18].

For the NGS RASopathy panel, the libraries were prepared using a custom HaloPlexHS panel (Agilent, Santa Clara, CA, USA), providing comprehensive coverage of the coding sequence and flanking intronic regions (+/−10 bp) of the *BRAF*, *CBL*, *HRAS*, *KRAS*, *LZTR1*, *MAP2K1*, *MAP2K2*, *NRAS*, *PPP1CB*, *PTPN11*, *RAF1*, *RIT1*, *RRAS*, *SHOC2*, *SOS1*, and *SOS2* genes. The enriched libraries were sequenced using a MiSeq sequencing platform (Illumina, San Diego, CA, USA). Sequencing data were processed and analyzed using a custom bioinformatics software pipeline. Reads were aligned to the GRCh37/hg19 reference genome by BWA (v.0.7.17). BAM files were sorted by SAMtools (v.1.7) and purged from duplicates using Mark Duplicates from the Picard suite (v.2.9.0). Mapped reads were locally realigned and base-quality-score recalibrated using GATK 3.8. Reads with mapping quality scores lower than 20 or with more than one-half nucleotides with quality scores less than 30 were filtered out. The GATK’s Haplotype Caller and ANNOVAR were used to identify and annotate single-nucleotide variants and indels. Bidirectional Sanger sequencing was performed using the ABI BigDye Terminator Sequencing Kit v.3.1 (ThermoFisher Scientific) and an ABI 3130 (ThermoFisher Scientific). 

## 3. Results

### 3.1. Systematic Review of the Literature

**Figure 1:**
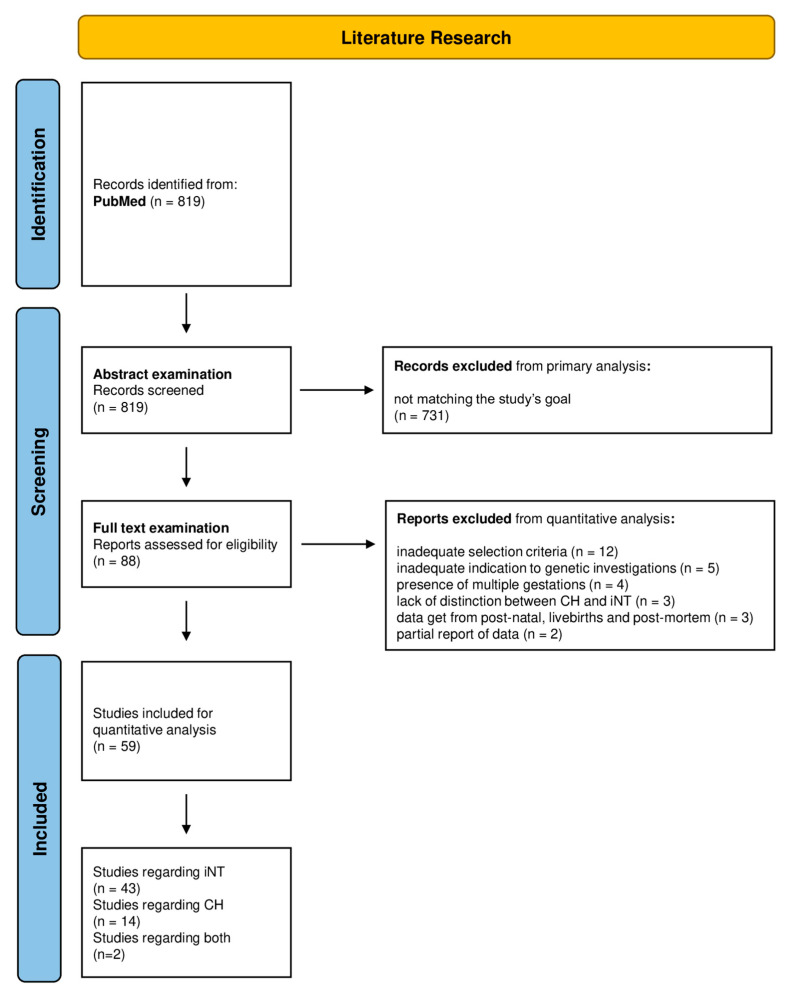
PRISMA flow chart of the systematic review and quantitative analysis.

The research results are presented according to the PRISMA guidelines workflow in Figure 1 [16].

The research conducted on PubMed provided 819 initial results. Of these, a total of 640 papers were excluded at abstract examination as not relevant to the research topic. In total, 179 papers remained and were later examined. Of these, 32 papers did not have complete available text, 22 were not in English and were secondary excluded. In total, 125 were ultimately retained for full-text examination. Of the remaining papers, 37 did not provide quantitative data, while 88 were examined for quantitative analysis. Twenty-nine papers were secondarily excluded from data analysis: 12 papers for inadequate selection criteria to invasive genetic testing [19,20,21,22,23,24,25,26,27,28,29,30]; in 5 papers, the indication to genetic testing was inadequate for incremental yield [31,32,33,34,35]; in 4 papers, it was not possible to distinguish multiple gestations [36,37,38,39]; 3 papers did not provide appropriate differentiation between hygroma and iNT [2,40,41]; 3 papers included data from postnatal, live births and postmortem examination [42,43,44]; 2 papers provided a partial report of data about fetuses’ malformations [45,46]. A total of 59 papers were adequate for quantitative analysis: 43 papers described the diagnostic application of karyotype, CMA, Rasopathy testing and/or ES in iNT cases (Table 1) [14,47,48,49,50,51,52,53,54,55,56,57,58,59,60,61,62,63,64,65,66,67,68,69,70,71,72,73,74,75,76,77,78,79,80,81,82,83,84,85,86,87,88]; 14 papers described their application in cystic hygroma (Table 2) [11,12,89,90,91,92,93,94,95,96,97,98,99,100], and 2 papers their application in both iNT and cystic hygroma (Table 1 and Table 2) [101,102]. Data concerning postnatal outcomes were retrieved from 17 papers for iNT (Table 3) [51,63,64,65,66,68,73,80,81,82,83,84,85,86,87,88,102,103] and from 10 papers for cystic hygroma (Table 4) [12,33,90,91,93,96,97,98,99,102].

The table illustrates data retrieved from the reference papers on the diagnostic yield of karyotype and the progressively incremental diagnostic yield of CMA in karyotype-negative cases, of RASopathy testing in karyotype- and CMA-negative cases, and ultimately of ES with iNT. For each reference, the cases were divided in categories based on the dimension of the NT and on whether the association with major fetal anomalies was reported or not. For the purpose of this review, chromosomal imbalances ≥ 10 Mb in size were considered karyotype-detectable, even if originally identified by CMA performed as a first-tier test. Following the same principle, variants identified in RASopathy genes by ES were classified under the “RASopathy panel” column.

The table illustrates data retrieved from the reference papers on the diagnostic yield of karyotype and the progressively incremental diagnostic yield of CMA (chromosomal microarray analysis) in karyotype-negative cases, of RASopathy testing in karyotype and CMA-negative cases, and ultimately of ES (exome sequencing) with cystic hygroma. For each reference, the cases were divided in categories based on whether the association with major fetal anomalies was reported or not. As stated in the previous section, the classification is based on the presence or putative absence of further anomalies at US examinations of the first and second trimester and before invasive genetic testing. The term “apparently isolated” identifies cases in which no other anomalies were documented at the time of genetic testing, so it does not indicate cases in which the anomaly appears as isolated throughout the whole pregnancy. For the purpose of this review, chromosomal imbalances ≥ 10 Mb in size were considered karyotype-detectable, even if originally identified by CMA performed as first-tier test. Following the same principle, variants identified in RASopathy genes by ES were classified under the “RASopathy panel” column.

The table illustrates data retrieved from the reference papers on the pregnancy and post-natal outcomes of fetuses with iNT. For each reference, the cases were divided in categories based on NT dimensions, on whether aneuploidies were excluded and whether the association with major fetal anomalies was reported or not.

The table illustrates data retrieved from the reference papers on the pregnancy and post-natal outcomes of fetuses with cystic hygroma. For each reference, the cases were divided into categories based on NT dimensions, on whether aneuploidies were excluded and whether the association with major fetal anomalies was reported or not.

### 3.2. Meta-Analysis

The results on the diagnostic yield of karyotype, and progressive incremental yield of CMA, RASopathy testing and exome sequencing (ES) iNT (2.5–2.9 mm, 2.5–3.4 mm, 3.0–3.4 mm, ≥2.5 mm, ≥3.0 mm, ≥3.5 mm) and cystic hygroma, either apparently isolated, associated with major fetal anomalies, or unspecified, are presented in Table 5.

### 3.3. Present Fetal Cohort

#### 3.3.1. Cystic Hygroma

Forty-seven out of the 96 fetuses were detected with isolated cystic hygroma (27 males and 20 females) (Table 6). Soft markers and other sonographic findings were noted: 2 ductus venosus agenesis, 1 hypoplastic nasal bone, 1 hyperechoic bowel, 2 absent nasal bone, 1 echoic cardiac focus and 1 fetus with single umbilical artery, ductus venosus agenesis and absent nasal bone.

Twenty-one of the fetuses (10 males, 11 females) were diagnosed with karyotype anomalies (44.68%): 12 constitutional trisomy of chromosome 21, 1 trisomy 21 mosaicism, 1 trisomy of the chromosome 13, 3 monosomy of X chromosome, 1 Klinefelter syndrome, 1 trisomy 21 with balanced autosomal translocation, 2 4p- (Wolf–Hirschhorn Syndrome).

The 26 cases showing normal standard karyotype underwent chromosomal microarray analysis and in 1 male fetus the 22q11.2 deletion syndrome was detected. In another case, a pathogenic copy number variation was identified.

In the remaining 25 fetuses, the multigene RASopathy panel was performed: in 3 cases (1 male, 2 females), a pathogenic variant was identified: c.179_181delTGG in *PTPN11* (NM_002834.5, MIM*176876), c.770C>T, p.(Ser257Leu) in *RAF1* (NM_002880.4, MIM*164760) and c.807_808delinsTT, p.(Gln269_His270delinsHisTyr) in *SHOC2* (NM_007373.4, MIM*602775) [104]. Four inherited VUSs were noted.

In this cohort, standard karyotype showed a detection rate of 44.68%. CMA presented an incremental diagnostic yield (over karyotype) of 3.8% and the incremental detection rate of the NGS RASopathy panel over CMA was 12%.

#### 3.3.2. Increased Nuchal Translucency

In 19 out of the 49 fetuses (Table 7), standard karyotype identified chromosomal anomalies (12/19 females, 63.12%), with a diagnostic yield of 38.78%, detailed below. No fetal pathogenic or likely pathogenic rearrangement was detected by CMA (0/30, 0%). The multigene RASopathy panel showed a detection rate of 0% (0–1/30).

**2.5–2.9 mm Nuchal Translucency**

In 6 out of the 49 fetuses, the NT ranged from 2.5 to 3 mm (two males, four females). Four fetuses were diagnosed with karyotype anomalies (three females, one male): 3 constitutional trisomy 21 and 1 Jacobs syndrome. CMA detected no copy number variations. The NGS RASopathy panel identifies in one fetus the paternally inherited c.643T>C, p.(Tyr215His) variant in *SOS1* (NM_005633.4, MIM*182530), classified as VUS.

In this cohort, standard karyotype showed a detection rate of 66.67%. CMA presented an incremental diagnostic yield (over karyotype) of 0% and the incremental detection rate of the NGS RASopathy panel over CMA was 0%.

**3.0–3.4 mm Nuchal Translucency**

In 18 out of the 49 fetuses, the NT measured 3.0–3.4 mm (11 males, 7 females). In one case, the fetus presented with hyperechoic cardiac focus and choroid plexus cysts; in a second case, hypoplastic nasal bone was noted. Eight fetuses were diagnosed with karyotype anomalies (three males, five females): 6 constitutional trisomy 21, 1 trisomy 18 mosaicism, 1 Klinefelter syndrome. CMA detected copy number variations in three cases, all classified as VUS. The NGS RASopathy panel did not reveal VUS/likely pathogenic/pathogenic variants.

In this cohort, standard karyotype showed a detection rate of 33.33%. CMA presented an incremental diagnostic yield (over karyotype) of 0% and the incremental detection rate of the NGS RASopathy panel over CMA was 0%.

**3.5–3.9 mm Nuchal Translucency**

In 8 out of the 49 fetuses, the NT measured 3.5–3.9 mm (four males, four females). Two fetuses, a male and a female, presented Trisomy 21. No other karyotype anomaly was identified. CMA did not detect rearrangements. The NGS RASopathy panel did not identify pathogenic variants related to the phenotype. The c.842del, p.(Pro281fs*) variant in *LZTR1* (NM_006767.4, MIM*600574) was identified in heterozygosity in a female fetus. The variant was not identified in the mother, but the father was not available for analysis.

In this cohort, standard karyotype showed a detection rate of 25.0%. CMA presented an incremental diagnostic yield (over karyotype) of 0% and the incremental detection rate of the NGS RASopathy panel over CMA was 0%.

**≥4.0 mm Nuchal Translucency**

In 16 out of the 49 fetuses, the NT measured equal to or more than 4.0 mm (nine males, seven females). Five fetuses (two males, three females) were diagnosed with karyotype anomalies: 2 constitutional trisomy of chromosome 21, 2 trisomy 18 and one with a large deletion of chromosome 3p. CMA detected one copy number variation, classified as VUS. The multigene RASopathy panel identified the maternally inherited heterozygous variant c.26G>C, p.(Gly9Ala) in *BRAF* (NM_004333.6, MIM*164757), classified as VUS.

In this cohort, standard karyotype showed a detection rate of 31.25%. CMA presented an incremental diagnostic yield (over karyotype) of 0% and the incremental detection rate of the NGS RASopathy panel over CMA was 0%.

## 4. Discussion

### 4.1. Physiopathology of Nuchal Fluid Collections

In physiological pregnancies, the NT increases in size with gestational age until about 13 gestational weeks, disappearing after the 14th week [105,106]. Similarly, iNT tends to reabsorb after this period [107], but in some cases it can persist or progress into other fetal fluid collections [108,109,110]. The regression of iNT before the 14th week is not an uncommon event and it has been observed in around 18% of iNT cases [88]. Despite the regression, those fetuses maintain higher risks than the normal population [88]. The presence of higher risk for genetic conditions and adverse outcome despite an apparently reassuring NT regression should be discussed in genetic counseling.

Some authors agree that it can be not easy to differentiate non-septated cystic hygroma and iNT. The first-trimester iNT may also include the initial findings of cystic hygroma, especially when a notch is seen in this area [40]. However, in both sonographic entities genetic testing plays a fundamental role in offering the couple an informed choice in decision-making of the ongoing pregnancy, to formulate the recurrence risks and to define the most appropriate management [111].

Fetal nuchal fluid collection can present as apparently isolated findings, be associated with concurrent malformations or occur as secondary lymphatic drainage anomalies due to cardiovascular malformations or skeletal dysplasia. In fetuses with aneuploidies, an alteration in the drainage of physiologically accumulated nuchal fluid can determine iNT or cystic hygroma. Underlying causes in aneuploid fetuses include cardiac malformations, primary abnormalities of lymphatic vessels and an altered composition of subcutaneous collagen [112,113]. In trisomic fetuses with iNT, increased levels of atrial and brain natriuretic peptide mRNA were identified, suggesting the presence of heart strain [113]. Interestingly, some studies reported that aneuploid fetuses with abnormal blood flow in the ductus venosus showed an umbilical cord diameter above the 95th centile for CRL, if compared to euploid fetuses at 11–14 weeks of gestation [114]. It can be secondary to abnormal ductus venosus flow during presystole, leading to venous congestion, transudation into the Wharton’s jelly and umbilical cord dilatation [115].

Some authors speculate that a slight iNT in male fetuses can represent a physiological gender-related characteristic in early development of the cardiovascular system and in the permeability of fetal skin. Testosterone production may reduce the epidermal thickness, lamellar body production and stratum corneum integrity [116] and the possibility of a favorable outcome was considered 1.8-fold higher in males than in females [117].

The definition of cystic hygroma is often reported ambiguously in the analyzed articles, which is why it is not possible to exclude selection bias of the fetuses included in the cohorts. Several authors defined that incomplete obstruction of lymphatic drainage, which can resolve spontaneously, may determine the formation of a non-septated cystic hygroma, while the complete obstruction may account for a septated cystic hygroma [118,119]. In some studies, the cohort was selected based on the presence of the septation, trying to make a further distinction within the category of cystic hygroma.

It is accepted that cystic hygroma is associated with high rates of aneuploidies, major structural anomalies, fetal demise, and poor outcome. Additionally, some studies reported that as the width increases, the odds of anomalies increase [12]. Interestingly, a paper demonstrated lower rates of chromosomal anomalies, and a higher number of good birth outcomes, in fetuses in which the cystic hygroma was detected with CRL measuring <45 mm, if compared to those detected later, without different prevalence of major malformations and intrauterine death among fetuses with normal karyotype [98]. However, these are data to be considered cautiously, as it would be necessary to know the causes for which the pregnant women performed this ultrasound before the scheduled time. Several authors observed that some cases with early disappearance of the cystic hygroma progressed to have normal outcomes [98,100]. It has been discussed that usually there is not a clear distinction between the “cystic hygroma” and the “iNT” phenotypes and that NT thickness may be the best independent predictor of aneuploidies [2].

iNT is detected in around 4.4% of euploid fetuses [10], and NT ≥ 3.0 mm, both in the unselected cohort and in high-risk populations, increased the risk of malformation almost 10-fold and the risk of miscarriage about 5-fold, in proportion to the NT width and without an association between iNT and perinatal death [103]. In the literature, the NT cut-off levels are extremely varied; therefore, the criteria for enrollment of cases are heterogeneous and not all papers could be included in the meta-analysis. Some studies recommended using MoM or delta-NT [120]; however, in most cases, for practicality, the absolute value of the measurement continued to be used. In fetuses with karyotype anomalies, further measurements of NT confirm the same alteration or reveal an increase in this thickness, while in fetuses with normal karyotyping the size generally decreases [62].

Many disorders are associated with iNT, suggesting that there may not be a single underlying mechanism for this condition: cardiovascular anomalies (also showing reduced diastolic function [121]), venous congestion in the head and neck from constriction of the fetal body, altered composition of the extracellular matrix, failure of lymphatic drainage, fetal anemia or hypoproteinemia, and infections (Parvovirus B19) [122]. Many proteins of the extracellular matrix are encoded on chromosomes 21, 18, or 13. In the nuchal skin of fetuses diagnosed with trisomy of chromosome 21, there is a substantial increase in hyaluronic acid and collagen type IV, which present hydrophilic properties leading to fluid accumulation [123].

iNT has been associated with fetal structural anomalies, in particular with cardiac malformations, and it has even been suggested that NT measurement can be used as a screening tool for fetal cardiac defects [124]. It is to be noted that, given the high prenatal prevalence of cardiac anomalies, some cases of apparently isolated iNT or cystic hygroma might actually underlie a cardiovascular defect [124]. For this reason, early echocardiographic assessment should be suggested in each fetus presenting with iNT or cystic hygroma. Obviously, some fetal anomalies may manifest at later stages of development and not in the gestational age in which NT screening is performed, which is why a careful sonographic morphological evaluation and an echocardiographic follow-up (even if no cardiovascular malformation has been detected) are required.

An unbiased evaluation of diagnostic yield of chromosomal, genomic or monogenic conditions in twin pregnancies is difficult to obtain, due to the increased risk of structural anomalies that could not be seen at the gestational age in which the invasive procedure is performed. iNT can also represent an early manifestation of the complications of placental vascular anastomoses in monochorionic twins [125,126], which would explain the higher incidence of chromosomal anomalies in dichorionic twins with one fetus detected with NT above the 95th centile than monochorionic twins [127].

Some authors showed that fetuses with isolated iNT and normal CMA present an increased risk for developing placental-related disorders in the following stages of the pregnancy [128].

### 4.2. Meta-Analysis on Genetic Testing and Present Fetal Cohort Considerations

The results of the meta-analysis highlight that, although increased nuchal translucency and cystic hygroma are both attributable to nuchal fluid collection, they appear to confer different risks for chromosomal, genomic and monogenic disorders.

In order to provide reliable information for prenatal counseling, we will discuss and compare meta-analysis data concerning cohorts with apparently isolated or explicitly associated iNT or cystic hygroma, as the unspecified/not reported cohorts might not reflect the actual presentation of cases in clinical practice.

In the reported cohorts, the indication to genetic testing is based on prenatal ultrasonographic findings. Ultrasound examination has inherent limitations, and a small but not negligible amount of anomalies might not be identified by initial ultrasound scans. For this reason, apparently isolated cases might have structural anomalies that become apparent only later in pregnancy or at birth. However, information from apparently isolated cases retrieved from the literature can be confidently proposed when counseling for fetuses with apparently isolated iNT or cystic hygroma in clinical practice, as both instances share the same limitations.

Standard karyotype revealed chromosomal anomalies in 48.39% (46.02–50.7) of fetuses with apparently isolated cystic hygroma included in the meta-analysis and in 44.68% of the fetuses included in the multicentric cohort. In the cohort we report, the incremental yield of CMA over karyotyping was 3.8%, while the NGS RASopathy panel presented a detection rate over CMA of 12%.

Regarding iNT, the parameters used are heterogeneous in the various cohorts; therefore, it was possible to carry out the meta-analysis by grouping the cases in the most frequently reported ranges, although these were partly overlapping with each other. NT ≥ 3.5 mm was the most common cut-off, followed by NT ≥ 3.0 mm. We scored the diagnostic yield of karyotyping, CMA, RASopathy testing and ES in the gathered cases by adopting these three cut-offs (all fetuses with NT ≥ 2.5, all fetuses with NT ≥ 3.0, all fetuses with NT ≥ 3.5), and also analyzed groups of fetuses with NT values in intervals within these cut-offs (2.5–2.9, 2.5–3.4, 3.0–3.4).

Fetuses with apparently isolated iNT that measured 2.5–2.9 mm showed chromosomal anomalies in 11.73% of cases involved in the meta-analysis and in 66.67% of fetuses enrolled in the cohort. To date, there are no univocal guidelines regarding the genetic investigations to be performed in the case of mild iNT; therefore, it can be complicated to define the selection criterion. It is possible that a proportion of pregnant women with iNT between 2.5 and 2.9 mm have performed screening testing (e.g., Non-invasive prenatal screening, combined screening of first trimester) resulting in high risk and are not included in these series as they refer to invasive investigation with a different indication. The data emerging from the meta-analysis regarding fetuses with this range of nuchal translucency can also be considered less realistic than the other ranges as they involve a smaller number of cases. CMA revealed a pathogenic rearrangement in 1.32% of cases (from only one article) and in no case of the present cohort.

Among the fetuses in which the apparently isolated iNT measured 3.0–3.4 mm included in the meta-analysis, 4.52% showed chromosomal anomalies, while in the retrospective cohort the detection rate of karyotype was 33.33% and the different diagnostic yield can easily be explained by the different sample size. CMA presented an incremental diagnostic yield over karyotype of 1.7% in the meta-analysis and of 0% in the present cohort.

It was also possible to group the data present in the literature on the basis of the minimum cut-off indicated for the iNT. In this way, overlaps have been created between the three groups.

In the meta-analysis, fetuses with apparently isolated NT ≥ 2.5 mm showed karyotype anomalies in 22.76% of cases and CMA presented an incremental detection rate of 2.35%. Fetuses with isolated NT ≥ 3 mm presented chromosomal anomalies in 14.36% of cases and CMA had an incremental detection rate of 3.89%. When the isolated NT measured at least 3.5 mm, the diagnostic yield of karyotyping was 34.35%, the incremental CMA detection rate was 4.1%, the incremental diagnostic rate of the RASopathy panel over CMA was 1.44%, while the incremental diagnostic rate of Clinical Exome Sequencing over the multigene RASopathy panel was 2.44%. Information for genetic counseling in apparently isolated iNT and cystic hygroma cases is provided in Table 8.

A limit of this methodology can be found in the different number of cases undergoing different tests due to a sequential and non-parallel approach that progressively reduces the sample size.

When considering associated cases, information is lacking for NT values between 2.5 and 3.5 mm. For cases with NT ≥ 3.5 mm and associated anomalies, chromosomal disorders are extremely probable, with a rate of aneuploidies as high as 79.13% [20], and and incremental yield of 13.56% for submicroscopic chromosomal imbalances identified by CMA. The frequency of RASopathies, 7.69%, also appears to be higher than in apparently isolated cases, being only 1.44%. The incremental yield of ES in cases with NT ≥ 3.5 mm and associated anomalies (22.92%) is also noteworthy, especially if compared to the 2.44% yield of ES in fetuses with apparently isolated iNT in the same ranges.

From the analysis of these data, it appears that the rate of chromosomal anomalies in fetuses with NT below 3.5 mm, which is instead a widely spread cut-off to define iNT, is far from negligible, and that even values between 2.5 and 2.9 present such risk. Interestingly, from these data it emerges that CMA presents a considerable diagnostic rate in the group of fetuses with NT ≥ 3.5 mm, while for lower values it has a marginal incremental yield over karyotype. Similarly, in the same group ES appears to show promising results and could be considered after a negative CMA result. For associated cases, the extremely high rates of chromosomal and genetic anomalies should be provided to consultants, and ES should be recommended in karyotype- and CMA- negative cases.

### 4.3. Long-Term Follow-Up after Pregnancies with Nuchal Fluid Collections

The evaluation of the pregnancy and, especially, post-natal outcomes of fetuses detected with iNT and cystic hygroma is heterogeneous in the literature, in methods and results, with a lack of proper cohorts. This is due to the marked difficulties of a years-long follow-up, and to the differences in the timing of the presentation of the possibly associated conditions. Terminations of pregnancy also negatively affect this field of research, as the recourse to termination varies greatly depending not only on ultrasound findings and genetic data, but also on cultural and law differences across countries. Data retrieved from the literature concerning pregnancy and post-natal outcomes are provided in Table 3 and Table 4. These evaluations are limited to euploid fetuses. In most cases, submicroscopic CNVs and RASopathies had not been excluded. Terminations of pregnancy were excluded from the count. It is possible that voluntary termination of pregnancy with severe presentations, even in euploid fetuses, skewed the results of pregnancy and post-natal outcomes towards an overall better prognosis [70,129]. There is an increased risk in iNT for miscarriage and intrauterine death (the conditions are often merged in the sources), the live-birth rate being 88.85% in the largest cohort (853 fetuses) of iNT ≥ 3.0 mm [68]. Some authors suggested the rate of miscarriage/intrauterine deaths to be proportional to the NT width, increasing from 1.6% when NT measured between the 95th and 99th centiles to about 20% for NT ≥ 6.5 mm [70,122]. The rate of fetal loss appears to be more consistent in cystic hygroma, with 7 out of 11 papers reporting a live birth rate below 50%. Perinatal deaths are only occasionally assessed and reported. One of the main concerns in ongoing pregnancies for prospective parents in these cases is the risk for neurodevelopmental anomalies. The evaluations have been performed in heterogeneous ways, usually without comparison with a control group, yielding different results [85]. A large study has documented a statistically significant association with intellectual disability and autism spectrum disorder with OR of 6.16 (95% CI, 1.51–25.0) and 2.48 (95% CI,1.02–5.99), respectively, in euploid fetuses with NT > 99th centile but not in lower increases [31]. Another interesting paper documented that the developmental quotient was significantly lower than controls, but still remained in the normal range at two years of age [130]. The association has not been extensively examined for cystic hygroma.

Both iNT and cystic hygroma can represent an early manifestation of different diseases, so each case must be scrupulously evaluated in order to define the indication to search for mutations in a panel of genes or perform whole exome sequencing. Major childhood morbidities, including malformations and genetic syndromes, are often reported in the literature, with a rate of 4.58% (28/611) and a 2.7% (10/370) in the largest cohorts [64,68]. It is to be noted that RASopathies can account for some of them [90]. Clearly, one of the limitations is that an extensive examination of all genes allows the identification of variants of unknown significance and can create difficulties in interpreting the results obtained [131,132]. Moreover, the fear of undetectable anomalies can induce anxiety even after negative genetic testing [79].

### 4.4. Future Perspectives

Interesting insights emerged from this analysis that could serve as a starting point for new studies. In particular, the path of genetic investigations which is usually performed sequentially was analyzed, defining the incremental diagnostic yield of the individual tests in fetuses with cystic hygroma and iNT.

Data concerning fetuses with NT measuring between 2.5 and 2.9 mm are less representative than those of the other ranges, due to a smaller sample size. It is possible that in this group there is a bias due to pregnant women who have performed screening tests resulting in high risk and have opted for voluntary termination of pregnancy before referring to genetic counseling or it is possible that they were not included in this study for a different indication to the invasive procedure.

The risk for chromosomal anomalies appears to be relevant even from NT values of 2.5 mm. The diagnostic yield of the CMA increases and becomes consistent above 3.5 mm.

Recently, promising studies have been performed in the literature regarding the diagnostic yield of the exome sequencing in the event of an ultrasound finding of fetal fluid collections, especially in the case of fetal hydrops, which can appear in the first trimester with an ultrasound picture attributable to iNT or cystic hygroma [133,134,135,136,137,138]. In up to 29% of cases, pathogenic variants had been identified, including RASopathies, inborn errors of metabolism, musculoskeletal disorders, lymphatic, cardiovascular, neurodevelopmental and hematologic diseases [133]. These insights may lead to consider the possibility of performing CMA and the RASopathy panel in parallel, in order to reach an earlier diagnosis. However, data concerning RASopathy panels or ES sequencing large cohorts of fetuses with isolated iNT, especially below 3.5 mm, are still lacking.

Future research should better explore the diagnostic rate of CMA at all iNT levels, and focus on the evaluation of monogenic conditions in fetuses presenting NT measurement between 2.5 and 2.9 mm or 2.5 and 3.4 mm. In this range, the rearrangements identified with CMA appear to be not frequent and the main chromosomal abnormalities responsible can also be identified by NIPT. Even if NIPS is recommended as the first choice for fetal diagnosis in pregnant women with smaller iNT who do not opt for invasive procedures [13], conventional cfDNA testing can miss the diagnosis in a high percentage of fetuses with iNT [38].

If the rate for submicroscopic chromosomal anomalies and for monogenic disorders is confirmed to be low, in that case, in countries where NIPS is offered as a public service it could be an approach to be offered to pregnant women with only slightly iNT, reducing unnecessary invasive procedures and parental anxiety.

Further studies may be decisive in determining the advantages of performing exome sequencing for iNT or cystic hygroma, also considering that these findings may represent characteristics of a fetal phenotype not yet evident in such an early gestational age.

Moreover, based on our experience, frequently in those cases in which the nuchal fluid collection remains an isolated finding and the genetic investigations are negative, an adequate follow-up is not performed after birth, which instead can prove to be important for the evaluation of the individual case.

## 5. Conclusions

Cystic hygroma and iNT, albeit belonging to the same phenotypic spectrum attributable to the fetal nuchal fluid collections, appear to present with a different prevalence of chromosomal, genomic and monogenic conditions both in the literature and in the present cohort. Karyotype abnormalities are the most frequent finding in these fetuses, even when the cut-off for iNT is a value below the common threshold of 3.5 mm or even below 3 mm, while rearrangements identified with CMA are more consistent when the NT is above 3.5 mm. The RASopathy panel detected pathogenic variants especially in fetuses diagnosed with cystic hygroma, with a diagnostic yield that is sometimes higher than in CMA.

Further studies are desirable, in order to define the most suitable diagnostic algorithm, determining the most appropriate path based on the ultrasound and dimensional characteristics, and also considering the possibility of performing exome sequencing.

## Figures and Tables

**Table 1:** Increased Nuchal Translucency and Genetic Testing-systematic review.

Nuchal Translucency						
Reference	Dimension (mm)	Association	Yield (Incremental)			
			Karyotype	CMA	RASopathy Panel	ES
**[47]**	2.5–2.9	unspecified	8/134 (5.97%)	3/126 (2.38%)	.	.
	3.0–3.4	unspecified	8/146 (5.48%)	2/138 (1.45%)	.	.
	3.5–4.4	unspecified	23/140 (16.43%)	7/117 (5.98%)	.	.
	4.5–5.4	unspecified	7/32 (21.88%)	0/25 (0.00%)	.	.
	5.5–6.4	unspecified	7/13 (53.85%)	1/6 (16.67%)	.	.
	≥6.5	unspecified	18/34 (52.94%)	2/16 (12.50%)	.	.
**[48]**	3.0–3.4	unspecified	15/110 (13.64%)	3/60 (5.00%)	.	.
	3.5–4.4	unspecified	28/83 (33.74%)	3/37 (8.11%)	.	.
	4.5–5.4	unspecified	18/40 (45.00%)	1/40 (2.50%)	.	.
	5.5–6.4	unspecified	12/30 (40.00%)	.	.	.
	6.5–7.4	unspecified	9/21 (42.86%)	.	.	.
	≥7.5	unspecified	21/35 (60.00%)	.	.	.
	≥4.5	unspecified	60/126 (47.62%)	1/40 (2.50%)	.	.
**[49]**	≥3.5	apparently isolated	.	.	2/73 (2.74%)	2/71 (2.82%)
**[50]**	≥2.5	unspecified	18/192 (9.38%)	.	.	.
	2.5–3.4	apparently isolated	.	2/119 (1.68%)	.	.
	2.5–3.4	associated	.	0/15 (0.00%)	.	.
	≥3.5	apparently isolated	.	1/43 (2.33%)	.	.
	≥3.5	associated	.	2/12 (16.67%)	.	.
**[51]**	≥3.5	apparently isolated	.	1/39 (2.56%)	.	.
**[52]**	≥3.5	apparently isolated	134/362 (37.02%)	1/229 (0.44%)	.	.
**[14]**	≥3.5	unspecified	123/226 (54.42%)	2/103 (1.94%)	3/103 (2.74%)	.
**[53]**	95ct–3.4	apparently isolated	18/114 (15.79%)	2/96 (2.01%)	.	.
	3.5–4.4	apparently isolated	30/150 (20.00%)	2/120 (1.67%)	.	.
	4.5–5.4	apparently isolated	16/55 (30.09%)	2/39 (5.13%)	.	.
	≥5.5	apparently isolated	29/55 (52.73%)	1/26 (3.85%)	1/22 (4.55%)	2/21 (9.52%)
**[54]**	3.0–3.4	apparently isolated	20/619 (32.31%)	9/599 (1.50%)	.	.
**[55]**	≥3.5	unspecified	57/175 (32.57%)	3/118 (2.54%)	.	.
**[56]**	2.5–3.4	unspecified	245/1372 (17.86%)	.	.	.
	3.5–4.4	unspecified	182/866 (21.02%)	.	.	.
	4.5–5.4	unspecified	77/282 (27.30%)	.	.	.
	5.5–6.4	unspecified	29/109 (26.61%)	.	.	.
	≥6.5	unspecified	27/91 (29.67%)	.	.	.
**[57]**	≥3.5	apparently isolated	.	.	0/111 (0.00%)	2/111 (1.80%)
	≥3.5	associated	.	.	7/91 (7.69%)	17/84 (20.24%)
**[58]**	3.0–3.4	apparently isolated	8/170 (4.70%)	3/162 (1.85%)	.	.
	≥3.5	apparently isolated	16/138 (42.11%)	3/122 (2.46%)	.	.
**[50]**	3.5–4.4	apparently isolated	16/76 (21.05%)	3/60 (5.00%)	.	.
	≥4.5	apparently isolated	27/56 (48.21%)	5/29 (17.24%)	.	.
**[60]**	2.5–3.4	unspecified	7/57 (12.28%)	1/49 (2.94%)	.	.
	3.5–4.4	unspecified	10/39 (25.64%)	1/29 (3.45%)	.	.
	4.5–5.4	unspecified	7/19 (36.84%)	0/12 (0.00%)	.	.
	≥5.5	unspecified	13/24 (54.17%)	0/11 (0.00%)	.	.
**[61]**	2.5–2.9	apparently isolated	10/86 (11.63%)	1/76 (1.32%)	.	.
	3.0–3.4	apparently isolated	11/73 (15.06%)	2/62 (3.23%)	.	.
	3.5–4.4	apparently isolated	9/50 (18.00%)	2/41 (4.88%)	.	.
	4.5–5.4	apparently isolated	10/21 (47.62%)	0/11 (0.00%)	.	.
	≥5.5	apparently isolated	7/11 (63.64%)	0/4 (0.00%)	.	.
**[62]**	≥95ct	unspecified	66/287 (23.00%)	.	.	.
**[63]**	≥3.5	unspecified	179/242 (73.96%)	.	.	.
**[64]**	≥95ct	unspecified	119/541 (22.00%)	.	.	.
**[65]**	≥3.5	unspecified	16/71 (22.54%)	.	.	.
**[66]**	≥3.0	unspecified	6/105 (5.71%)	.	.	.
**[67]**	≥2.5	unspecified	18/122 (14.75%)	.	.	.
**[68]**	≥3	unspecified	224/1058 (21.17%)	.	.	.
	3.0–3.4	unspecified	65/676 (9.62%)	.	.	.
	3.5–4.4	unspecified	51/208 (24.52%)	.	.	.
	4.5–5.4	unspecified	29/67 (43.28%)	.	.	.
	5.5–6.4	unspecified	26/37 (70.27%)	.	.	.
	≥6.5	unspecified	53/70 (75.71%)	.	.	.
**[69]**	95ct–3.4	unspecified	124/894 (13.87%)	8/770 (1.04%)	.	.
	≥3.5	unspecified	436/1007 (43.30%)	30/571 (5.25%)	.	.
	3.5–4.9	unspecified	138/492 (28.05%)	16/354 (4.52%)	.	.
	5.0–6.4	unspecified	113/199 (56.78%)	7/86 (8.14%)	.	.
	6.5–7.9	unspecified	93/155 (60.00%)	5/62 (8.06%)	.	.
	≥8.0	unspecified	92/162 (56.79%)	2/70 (2.86%)	.	.
**[101]**	≥3	unspecified	10/120 (8.33%)	.	.	.
**[70]**	95ct–3.4	unspecified	40/263 (15.21%)	.	.	.
	3.5–4.4	unspecified	32/169 (18.93%)	.	.	.
	4.5–5.4	unspecified	37/79 (46.84%)	.	.	.
	5.5–6.4	unspecified	33/52 (63.46%)	.	.	.
	≥6.5	unspecified	55/85 (64.71%)	.	.	.
**[71]**	≥3.5	apparently isolated	.	5/34 (14.70%)	.	.
	≥3.5	associated	.	3/16 (18.75%)	1/13 (7.69%)	5/12 (41.67%)
**[102]**	≥3.0	unspecified	8/46 (17.39%)	.	.	.
	3.0–3.9	unspecified	4/35 (11.43%)	.	.	.
	4.0–4.9	unspecified	2/7 (28.57%)	.	.	.
	5.0–5.9	unspecified	1/3 (33.33%)	.	.	.
	≥6.0	unspecified	1/1 (100.00%)	.	.	.
**[72]**	3.5–4.4	unspecified	.	4/343 (1.17%)	.	.
	4.5–5.4	unspecified	.	3/124 (2.42%)	.	.
	5.5–6.4	unspecified	.	4/73 (5.48%)	.	.
	≥6.5	unspecified	.	0/59 (0.00%)	.	.
**[73]**	≥99ct	unspecified	94/221 (42.53%)	1/106 (0.94%)	.	.
**[74]**	≥3.0	apparently isolated	21/108 (19.44%)	9/87 (10.34%)	.	.
	3.0–3.9	apparently isolated	12/81 (14.81%)	6/69 (8.70%)	.	.
	≥4.0	apparently isolated	9/27 (33.33%)	3/18 (16.67%)	.	.
**[75]**	≥3.0	unspecified	172/775 (22.19%)	4/256 (1.56%)	.	.
**[76]**	≥3.5	apparently isolated	.	1/172 (0.58%)	.	.
**[77]**	95ct–3.4	unspecified	507/7109 (7.13%)	.	.	.
	3.5–4.4	unspecified	423/2101 (20.13%)	.	.	.
	4.5–5.4	unspecified	321/707 (45.40%)	.	.	.
	5.5–6.4	unspecified	219/437 (50.11%)	.	.	.
	6.5–7.4	unspecified	218/309 (70.55%)	.	.	.
	7.5–8.4	unspecified	148/209 (70.81%)	.	.	.
	8.5–9.4	unspecified	126/168 (75.00%)	.	.	.
	9.5–10.4	unspecified	74/88 (84.09%)	.	.	.
	10.5–11.4	unspecified	45/64 (70.31%)	.	.	.
	≥11.5	unspecified	87/123 (70.73%)	.	.	.
**[78]**	≥3.5	apparently isolated	.	3/269 (1.12%)	.	.
	≥3.5	associated	.	3/31 (9.68%)	.	.
**[79]**	≥6.5	unspecified	60/84 (71.43%)	.	.	.
	5.5–6.4	unspecified	21/37 (56.76%)	.	.	.
**[80]**	≥3.5	unspecified	164/303 (54.13%)	.	.	.
**[81]**	≥3.5	unspecified	123/222 (55.41%)	.	.	.
	≥3.5	apparently isolated	32/107 (29.91%)	.	.	.
	≥3.5	associated	91/115 (79.13%)	.	.	.
**[82]**	≥95ct	unspecified	154/393 (39.19%)	.	.	.
	95ct–3.4	unspecified	31/170 (18.24%)	.	.	.
	3.5–4.4	unspecified	29/81 (35.80%)	.	.	.
	4.5–5.4	unspecified	23/42 (54.76%)	.	.	.
	5.5–6.4	unspecified	15/23 (65.22%)	.	.	.
	≥6.5	unspecified	56/77 (72.73%)	.	.	.
**[83]**	≥95ct	unspecified	37/186 (19.89%)	.	.	.
	95ct–3.4	unspecified	10/92 (10.87%)	.	.	.
	3.5–4.4	unspecified	6/50 (12.00%)	.	.	.
	4.5–5.4	unspecified	4/12 (33.33%)	.	.	.
	5.5–6.4	unspecified	7/15 (46.67%)	.	.	.
	≥6.5	unspecified	10/17 (58.82%)	.	.	.
**[84]**	2.5–4.4	unspecified	29/33 (87.88%)	.	.	.
	4.5–6.4	unspecified	8/8 (100.00%)	.	.	.
	≥6.5	unspecified	11/13 (84.62%)	.	.	.
**[85]**	≥99ct	unspecified	64/248 (25.81%)	.	.	.
**[86]**	≥4.0	unspecified	71/160 (44.38%)	.	.	.
**[87]**	≥6.5	unspecified	89/120 (74.17%)	.	.	.
**[88]**	≥95ct	unspecified	44/147 (29.93%)	.	.	.

**Table 2:** Cystic Hygroma and Genetic Testing-systematic review.

Cystic Hygroma					
Reference	Association	Yield (Incremental)			
		Karyotype	CMA	RASopathy Panel	ES
**[89]**	unspecified	15/28 (53.50%)	.	.	.
**[90]**	apparently isolated	25/50 (50.00%)	.	.	.
	associated	13/22 (59.09%)	.	.	.
**[91]**	unspecified	67/132 (50.76%)	.	.	.
**[92]**	unspecified	122/185 (65.95%)	1/40 (2.50%)	6/15 (40.00%)	.
**[93]**	apparently isolated	3/10 (30.00%)	.	.	.
	associated	13/20 (65.00%)	.	.	.
**[94]**	unspecified	55/85 (64.70%)	.	.	.
**[95]**	unspecified	20/37 (54.05%)	.	.	.
**[11]**	unspecified	18/50 (36.00%)	.	.	.
**[101]**	unspecified	13/27 (48.15%)	.	.	.
**[102]**	unspecified	13/30 (43.33%)	.	.	.
	unspecified	0/1 (0.00%)	.	.	.
	unspecified	1/3 (33.33%)	.	.	.
	unspecified	4/4 (100.00%)	.	.	.
	unspecified	11/22 (50.00%)	.	.	.
**[96]**	apparently isolated	12/21 (57.14%)	.	.	.
	associated	13/21 (61.90%)	.	.	.
**[97]**	apparently isolated	5/12 (41.67%)	.	.	.
	associated	7/14 (50.00%)	.	.	.
**[12]**	unspecified	400/729 (54.87%)	.	.	.
**[98]**	unspecified	128/194 (65.98%)	.	.	.
**[99]**	unspecified	28/69 (40.58%)	.	.	.
**[100]**	unspecified	45/100 (45.00%)	.	.	.

**Table 3:** Increased Nuchal Translucency and Prenatal/Postnatal Outcome-systematic review.

Nuchal Translucency								
Reference	Dimension (mm)	Association	Miscarriages	Intrauterine Deaths	Live Births	Perinatal Death	Intellectual Disability	Major Childhood Morbidity
**[52]**	≥3.5	apparently isolated-euploid	0/20 (0.00%)	1/20 (5.00%)	19/20 (95.00%)	1/19 (5.26%)	3/19 (15.79%)	3/19 (15.79%)
**[63]**	≥3.5	unspecified-euploid	.	5/33 (15.15%)	28/33 (84.85%)	.	.	.
**[64]**	≥3.5	unspecified-euploid	4/420 (0.95%)	.	.	.	10/270 (3.70%)	10/370 (2.70%)
**[65]**	≥3.5	unspecified-euploid	6/52 (11.54%)	.	46/52 (88.46%)	.	.	6/46 (13.04%)
**[66]**	≤3.0	unspecified	45/1395 (3.23%)	19/1395 (1.36%)	1305/1395 (93.55%)	.	.	.
	≥3.0	unspecified	22/105 (20.95%)	15/105 (14.29%)	33/105 (31.43%)	.	.	.
	3.0–4.0	unspecified	10/52 (19.23%)	5/52 (9.62%)	28/52 (53.85%)	.	.	.
	4.0–5.0	unspecified	10/33 (30.30%)	7/33 (21.21%)	5/33 (15.15%)	.	.	.
	5.0–6.0	unspecified	2/15 (13.33%)	3/15 (20.00%)	0/15 (0.00%)	.	.	.
	≥6.0	unspecified	0/5 (0.00%)	0/5 (0.00%)	0/5 (0.00%)	.	.	.
**[68]**	≥3.0	associated-euploid	.	.	741/834 (88.85%)	.	.	43/741 (5.80%)
	3–3.4	associated-euploid	.	.	562/611 (91.98%)	.	.	28/562 (4.98%)
	3.5–4.4	associated-euploid	.	.	141/157 (89.81%)	.	.	7/141 (4.96%)
	4.5–5.4	associated-euploid	.	.	30/38 (78.95%)	.	.	2/30 (6.67%)
	5.5–6.4	associated-euploid	.	.	5/11 (45.45%)	.	.	4/5 (80.00%)
	≥6.5	associated-euploid	.	.	3/17 (17.65%)	.	.	2/3 (66.76%)
**[102]**	≥3.0	unspecified	.	0/46 (0.00%)	31/46 (67.39%)	3/31 (9.68%)	.	3/31 (9.68%)
**[73]**	≥99ct	associated-euploid	.	4/36 (11.11%)	13/36 (36.11%)	1/6 (16.67%)	.	6/13 (46.15%)
	≥99ct	apparently isolated-euploid	2/70 (2.86%)	1/70 (1.73%)	63/70 (90.00%)	.	.	3/63 (4.76%)
**[103]**	≥95ct	unspecified-euploid	23/625 (3.68%)	.	.	.	.	.
**[80]**	≥3.5	unspecified-euploid	5/139 (3.60%)	1/139 (0.72%)	110/139 (79.14%)	.	.	7/110 (6.36%)
	3.5–4.4	unspecified-euploid	2/86 (2.33%)	1/86 (1.16%)	77/86 (89.53%)	.	.	3/77 (3.90%)
	4.5–5.4	unspecified-euploid	0/28 (0.00%)	0/28 (0.00%)	20/28 (71.43%)	.	.	2/20 (10.00%)
	5.5–6.4	unspecified-euploid	1/12 (8.33%)	0/12 (0.00%)	7/12 (58.33%)	.	.	1/7 (14.29%)
	≥6.5	unspecified-euploid	2/13 (15.38%)	0/13 (0.00%)	6/13 (46.15%)	.	.	1/6 (16.67%)
**[82]**	≥95ct	unspecified	.	9/239 (3.77%)	210/239 (87.87%)	.	.	10/210 (4.76%)
	95ct–3.4	unspecified	.	2/139 (1.44%)	135/139 (97.12%)	.	.	2/135 (1.48%)
	3.5–4.4	unspecified	.	2/52 (3.85%)	46/52 (88.46%)	.	.	2/46 (4.35%)
	4.5–5.4	unspecified	.	2/19 (10.53%)	16/19 (84.21%)	.	.	1/16 (6.25%)
	5.5–6.4	unspecified	.	0/8 (0.00%)	4/8 (50.00%)	.	.	0/4 (0.00%)
	≥6.5	unspecified	.	3/21 (14.29%)	9/21 (42.86%)	.	.	5/9 (55.56%)
**[83]**	≥95ct	unspecified	7/149 (4.70%)	.	110/149 (73.83%)	.	.	.
	95ct–3.4	unspecified	1/82 (1.22%)	.	71/82 (86.59%)	.	.	.
	3.5–4.4	unspecified	2/43 (4.65%)	.	35/43 (81.40%)	.	.	.
	4.5–5.4	unspecified	0/7 (0.00%)	.	2/7 (28.57%)	.	.	.
	5.5–6.4	unspecified	3/8 (37.50%)	.	1/8 (12.50%)	.	.	.
	≥6.5	unspecified	1/19 (5.26%)	.	1/19 (5.26%)	.	.	.
**[84]**	2.5–4.4	unspecified	4/33 (12.12%)	0/33 (0.00%)	20/33 (60.61%)	.	.	.
	4.5–6.4	unspecified	1/8 (12.5%)	0/8 (0.00%)	1/8 (12.50%)	.	.	.
	≥6.5	unspecified	2/13 (15.38%)	4/13 (30.77%)	1/13 (7.69%)	.	.	.
**[85]**	≥99ct	unspecified-euploid	1/179 (0.56%)	5/179 (2.79%)	162/179 (90.50%)	.	.	18/162 (11.11%)
**[86]**	≥4.0	unspecified	.	2/89 (2.25%)	68/89 (76.40%)	.	4/64 (6.25%)	4/68 (5.88%)
**[87]**	≥6.5	unspecified-euploid	6/27 (22.22%)	.	8/27 (29.63%)	.	.	.
**[88]**	≥95ct	unspecified-euploid	.	10/103 (9.71%)	87/103 (84.47%)	.	.	10/87 (11.49%)

**Table 4:** Cystic Hygroma and Prenatal/Postnatal Outcome-systematic review.

Cystic Hygroma							
Reference	Association	Miscarriages	Intrauterine Deaths	Live Births	Perinatal Death	Intellectual Disability	Major Childhood Morbidity
**[90]**	unspecified-euploid	2/34 (5.88%)	2/34 (5.88%)	18/34 (52.94%)	0/18 (0.00%)	.	2/18 (11.11%)
**[91]**	unspecified-euploid	.	5/36 (13.89%)	31/36 (86.11%)	1/31 (3.23%)	1/31 (3.23%)	8/31 (25.81%)
**[93]**	associated-euploid	.	1/3 (33.33%)	2/3 (66.66%)	.	.	.
	apparently isolated-euploid	.	.	6/6 (100.00%)	.	.	.
**[102]**	unspecified	.	5/30 (16.67%)	7/30 (23.33%)	4/7 (57.14%)	.	2/7 (28.57%)
**[96]**	unspecified-euploid	.	.	7/17 (41.18%)	.	.	.
**[97]**	apparently isolated-euploid	1/5 (20.00%)	.	3/5 (60.00%)	.	.	1/3 (33.33%)
	associated-euploid	3/7 (42.86%)	1/7 (14.29%)	1/7 (14.29%)	.	.	1/1 (100.00%)
**[12]**	unspecified	106/295 (14.29%)	.	180/295 (24.26%)	9/180 (5.00%)	.	.
**[98]**	unspecified-euploid	7/66 (10.61%)	.	27/66 (40.91%)	.	.	.
**[99]**	unspecified	5/41 (12.20%)	.	12/41 (29.27%)	.	.	.
**[100]**	unspecified	.	54/85 (63.53%)	31/85 (36.47%)	.	.	6/31 (19.35%)

**Table 5:** Increased Nuchal Translucency and Cystic Hygroma and Genetic testing-meta-analysis.

Nuchal Translucency						
Dimensions	Association	Yield (Incremental)				REF[Karyo][CMA][RAS][ES]
		Karyotype	CMA	RASopathy Panel	ES	
2.5–2.9	unspecified	8/134 5.97%	11/896 1.23% (1.17–1.29)	.	.	[61]; [61]; [.]; [.]
	apparently isolated	10/86 11.73%	1/76 (1.32%)	.	.	[47,69]; [47]; [.]; [.]
	associated	.	.	.	.	[.]; [.]; [.]; [.]
2.5–3.4	unspecified	964/9957 9.68% (9.60–9.76)	9/819 1.10% (1.05–1.15)	.	.	[56,60,69,77,82,83]; [60,69]; [.]; [.]
	apparently isolated	39/273 14.29% (14.07–14.51)	4/215 1.86% (1.82–1.90)	.	.	[53,61]; [50,53]; [.]; [.]
	associated	.	0/15 0.00%	.	.	[.]; [50]; [.]; [.]
3.0–3.4	unspecified	88/932 9.44% (9.18–9.70)	5/198 2.53% (2.18–2.88)	.	.	[47,48,68]; [47,48]; [.]; [.]
	apparently isolated	39/862 4.52% (4.09–4.95)	14/823 1.70% (1.64–1.76)	.	.	[54,58,61]; [54,58,61]; [.]; [.]
	associated	.	.	.	.	[.]; [.]; [.]; [.]
≥2.5	unspecified	4393/19177 22.91% (22.60–23.22)	61/2098 2.91% (2.88–2.94)	.	.	[47,50,56,60,62,64,67,69,70,77,82,83,84,88]; [47,50,60,69]; [.]; [.]
	apparently isolated	140/615 22.76% (22.46–23.06)	15/637 2.35% (2.32–2.38)	.	.	[53,61]; [50,53,61]; [.]; [.]
	associated	2/134 1.49%	2/27 (7.41%)	.	.	[50]; [50]; [.]; [.]
≥3.0	unspecified	616/2788 22.09% (21.74–22.44)	23/695 3.31% (3.18–3.44)	.	.	[47,48,66,68,75,101,102]; [47,48,75]; [.]; [.]
	apparently isolated	82/571 14.36% (13.68–15.04)	19/489 3.89% (3.50–4.28)	.	.	[58,61,74]; [58,61,74]; [.]; [.]
	associated	.	.	.	.	[.]; [.]; [.]; [.]
≥3.5	unspecified	3680/9204 39.98% (39.64–40.32)	59/1581 3.73% (3.64–3.82)	3/103 (2.91%)	.	[47,48,55,56,60,63,65,68,69,70,77,80,81,82,83]; [47,48,55,60,69,72]; [14]; [.]
	apparently isolated	449/1307 34.35% (33.66–35.04)	56/1367 4.1% (3.50–4.70)	3/108 1.44% (1.15–1.73)	5/205 2.44% (2.26–2.62)	[14,52,53,58,59,61,81]; [14,50,51,52,53,58,59,61,71,76,78]; [49,53,57]; [49,53,57]
	associated	91/115 79.13%	8/59 13.56% (12.32–14.80)	8/104 (7.69%)	22/96 22.92% (19.85–25.99)	[81]; [50,71,78]; [57,71]; [57,71]
**Cystic Hygroma**						
**Association**		**Yield (incremental)**				**REF[Karyo][CMA][RAS][ES]**
		**Karyotype**	**CMA**	**RASopathy panel**	**ES**	
unspecified		927/1666 55.64% (55.18–56.10)	1/40 2.50%	6/15 (40.00%)	.	[11,12,89,91,92,94,95,99,100,101,102]; [92]; [92]; [.]
apparently isolated		45/93 48.39% (46.02–50.76)	.	.	.	[90,93,96,97]; [.]; [.]; [.]
associated		46/80 57.50% (55.16–59.84)	.	.	.	[90,93,96,97]; [.]; [.]; [.]

CMA: chromosomal microarray analysis; RAS: RASopathy panel; ES: exome sequencing.

**Table 6:** Present fetal cohort–cystic hygroma.

ID	Sex	Growth	Soft Marker (s)	Karyotype	CMA	RASopathy Panel
1	F	-	-	46,XX	N	N
2	F	-	-	46,XX	N	*LZTR1* c.2173_2174insA; p.(Arg725Glnfs*57) mat
3	F	-	inverted DV flow	mos 47,XX,+21[27]/46,XX [3]	.	.
4	F	-	-	47,XX,+21	.	.
5	F	-	-	46,XX	N	*SOS1* c.755T>C; p.(Ile252Thr) VUS pat
6	F	-	-	46,XX	N	N
7	F	biometry <5°c	absent nasal bone	46,XX	N	*RAF1*: c.770C>T; p.(Ser257Leu) dn
8	F	-	-	45,X	.	.
9	F	-	-	46,XX	N	N
10	F	-	-	46,XX	N	N
11	F	-	-	46,XX	N	N
12	F	-	-	46,XX	N	N
13	F	-	-	46,XX	N	*PTPN11* c.179_181delTGG
14	F	-	absent nasal bone	47,XX,+21	.	.
15	F	-	-	47,XX,+21,t(2;8)(q33;q22)	.	.
16	F	-	-	47,XY,+21	.	.
17	F	-	echoic cardiac focus	47,XX,+21	.	.
18	F	-	-	45,X	.	.
19	F	-	-	47,XX,+21	.	.
20	F	-	-	45,X	.	.
21	M	-	DVA	46,XY	N	*SHOC2* c.807_808delinsTT; p.(Gln269_His270delinsHisTyr)
22	M	-	hypoplastic nasal bone	46,XY	N	*SOS2* c.1709C>G; p.(Pro570Arg) VUSmat
23	M	-	-	47,XY,+21	n.p.	n.p.
24	M	-	-	46,XY	N	N
25	M	-	-	46,XY	N	N
26	M	-	-	46,XY	22q11.21(18894835_21464119)x1	
27	M	-	-	46,XY	N	N
28	M	-	DVA	46,XY der(4)t(4;7)(4p16.3;7p22.3)	4p16.3(71552_3872380)x1 7p22.3p22.1(42976_6870943)x3	
29	M	-	hyperechoic bowel	46,XY	N	N
30	M	-	-	46,XY	N	N
31	M	-	-	46,XY	N	*SOS1* c.643T>C; p.(Tyr215His) mat
32	M	-	-	46,XY	N	N
33	M	-	-	46,XY	N	N
34	M	-	-	47,XXY	.	.
35	M	-	-	47,XY,+21	.	.
36	M	-	-	46,XY,del(4)(p15.2)dn	.	.
37	M	-	-	47,XY,+21	.	.
38	M	-	-	47,XY,+13	.	.
39	M	-	-	47,XY,+21	.	.
40	M	-	-	47,XY,+21	.	.
41	M	-	-	46,XY	N	N
42	M	-	-	46,XY	N	N
43	M	-	-	46,XY	N	N
44	M	-	-	46,XY	17q23.2(60033664_60251568)x3 mat	N
45	M	-	SUA, DVA, absent nasal bone	47,XY,+21	.	.
46	M	-	-	46,XY	N	N
47	M	-	absent nasal bone	47,XY,+21	.	.

CMA: chromosomal microarray analysis; N: negative; F: female; M: male; DV: ductus venosus; DVA: ductus venosus agenesis; SUA: single umbilical artery.

**Table 7:** Present fetal cohort–increased nuchal translucency.

ID	Sex	NT (mm)	Soft Marker (s)	Karyotype	CMA	RASopathy Panel
**NT 2.5–2.9 mm**						
**1**	**F**	**2.6**	**-**	**47,XX,+21**	.	.
2	M	2.6	-	46,XY	N	*SOS1* c.643T>C (het, mat)
**3**	**F**	**2.7**	**-**	**47,XX,+21**	.	.
**4**	**F**	**2.8**	**-**	**47,XX,+21**	.	.
5	F	2.8	-	46,XX	N	N
**6**	**M**	**2.9**	**-**	**47,XYY**	.	.
**NT 3.0–3.4 mm**						
**7**	**F**	**3.0**	**-**	**47,XX,+21**	.	.
9	F	3.0	-	46,XX	N	N
**10**	**F**	**3.0**	**-**	**47,XX,+21**	.	.
11	M	3.0	-	46,XY	10p13p12.33(16786323_17726950)x3 mat	.
**12**	**M**	**3.0**		**47,XXY**	10q21.3(68735256_68938523)x3 pat 22q12.2(29911595_30141397)x3 mat	.
13	M	3.0	hypoplastic nasal bone	46,XY	N	N
**14**	**M**	**3.1**	**-**	**47,XY,+21**	.	.
**15**	**F**	**3.1**	**-**	**47,XX,+21**	.	.
**16**	**F**	**3.2**	**-**	**mos 47,XX,+18**[58]**/46,XX** [20]	.	.
17	M	3.2	-	46,XY	N	N
18	M	3.2	-	N	N	N
19	M	3.3	-	46,XY	N	N
20	M	3.3	-	46,XY	N	N
**21**	**F**	**3.3**	**-**	**47,XX,+21**	.	.
22	M	3.3	-	46,XY	N	N
23	M	3.3	Choroid plexus cyst, hyperechoic cardiac focus	46,XY	N	N
**24**	**M**	**3.3**	**-**	**47,XY,+21**	.	.
25	F	3.4	-	46,XX	3p26.3(270649_920375)x3mat	N
**NT 3.5–3.9 mm**						
26	F	3.5	-	46,XX	N	N
**27**	**M**	**3.5**	**-**	**47,XY,+21**	.	.
28	M	3.6	-	46,XY	N	N
29	F	3.6	-	46,XX	N	N
30	M	3.6	-	N	N	N
31	M	3.8	-	N	N	N
**32**	**F**	**3.9**	**-**	**47,XX,+21**	.	.
33	F	3.9	-	46,XX	N	*LZTR1* c.842del (het, n/a)
**NT ≥ 4.0 mm**						
34	M	4.0	-	46,XY	N	N
35	M	4.0	-	46,XY	N	N
36	M	4.0	-	46,XY,inv(19)(p13.3q13.2)mat	N	N
37	M	4–0	-	46,XY	N	N
38	F	4.1	-	46,XX	N	N
**39**	**M**	**4.2**	**-**	**47,XY,+21**	.	.
40	M	4.2	-	46,XY	N	N
41	F	4.3	-	N	N	N
**42**	**F**	**4.4**	**-**	**47,XX,+21**	.	.
**43**	**F**	**4.7**	**-**	**46,XX,del(3)(p25)**	.	.
44	F	4.7	-	46,XX	N	*BRAF* c.26G>C (het, mat)
45	M	5.3	-	46,XY	N	N
**46**	**M**	**5.7**	**-**	**47,XY,+18**	.	.
**47**	**F**	**5.8**	**-**	**47,XX,+18**	.	.
48	F	7.0	-	46,XX	N	N
49	M	7.7	-	46,XY	15q11.2(22784523_23179948)x1	N

NT: nuchal translucency; CMA: chromosomal microarray analysis; N: negative; F: female; M: male.

**Table 8:** Apparently isolated iNT and cystic hygroma: summary of the meta-analysis findings.

	Apparently Isolated iNT			Apparently Isolated Cystic Hygroma
	2.5–2.9 mm	3–3.4 mm	≥3.5 mm	
**Karyotype**	11.73%	4.52%	34.35%	48.39%
**CMA**	1.32%	1.7%	4.1%	
**RASopathy panel**			1.44%	
**ES**			2.44%	

## Data Availability

The data presented in this study are available in the articles cited in the References section.
